# Development and characterization of a Yucatan miniature biomedical pig permanent middle cerebral artery occlusion stroke model

**DOI:** 10.1186/2040-7378-6-5

**Published:** 2014-03-23

**Authors:** Simon R Platt, Shannon P Holmes, Elizabeth W Howerth, Kylee Jo J Duberstein, C Robert Dove, Holly A Kinder, Emily L Wyatt, Amie V Linville, Vivian W Lau, Steven L Stice, William D Hill, David C Hess, Franklin D West

**Affiliations:** 1 Regenerative Bioscience Center, University of Georgia, Athens, GA 30602, USA; 2 Department of Small Animal and Surgery, University of Georgia, Athens, GA 30602, USA; 3 Department of Veterinary Biosciences & Diagnostic Imaging, University of Georgia, Athens, GA 30602, USA; 4 Department of Pathology, University of Georgia, Athens, GA 30602, USA; 5 Department of Animal and Dairy Science, University of Georgia, Athens, GA 30602, USA; 6 Department of Neurology, Georgia Regents University, Augusta, GA 30912, USA; 7 Department of Cellular Biology & Anatomy, Georgia Regents University, Augusta, GA 30912, USA

**Keywords:** Stroke model, Pig, Magnetic resonance imaging

## Abstract

**Background:**

Efforts to develop stroke treatments have met with limited success despite an intense need to produce novel treatments. The failed translation of many of these therapies in clinical trials has lead to a close examination of the therapeutic development process. One of the major factors believed to be limiting effective screening of these treatments is the absence of an animal model more predictive of human responses to treatments. The pig may potentially fill this gap with a gyrencephalic brain that is larger in size with a more similar gray-white matter composition to humans than traditional stroke animal models. In this study we develop and characterize a novel pig middle cerebral artery occlusion (MCAO) ischemic stroke model.

**Methods:**

Eleven male pigs underwent MCAO surgery with the first 4 landrace pigs utilized to optimize stroke procedure and 7 additional Yucatan stroked pigs studied over a 90 day period. MRI analysis was done at 24 hrs and 90 days and included T2w, T2w FLAIR, T1w FLAIR and DWI sequences and associated ADC maps. Pigs were sacrificed at 90 days and underwent gross and microscopic histological evaluation. Significance in quantitative changes was determined by two-way analysis of variance and post-hoc Tukey’s Pair-Wise comparisons.

**Results:**

MRI analysis of animals that underwent MCAO surgery at 24 hrs had hyperintense regions in T2w and DWI images with corresponding ADC maps having hypointense regions indicating cytotoxic edema consistent with an ischemic stroke. At 90 days, region of interest analysis of T1 FLAIR and ADC maps had an average lesion size of 59.17 cc, a loss of 8% brain matter. Histological examination of pig brains showed atrophy and loss of tissue, consistent with MRI, as well as glial scar formation and macrophage infiltration.

**Conclusions:**

The MCAO procedure led to significant and consistent strokes with high survivability. These results suggest that the pig model is potentially a robust system for the study of stroke pathophysiology and potential diagnostics and therapeutics.

## Introduction

Pre-clinical animal stroke models are a critical component of evaluating the evolution and pathophysiological mechanisms of cerebral ischemia, potential diagnostics and therapeutics [1-4]. Rodent models have been heavily relied upon with the development of numerous permanent and temporary ischemic injury approaches ranging from simple cauterization or ligation methods to more complex embolic or photothrombotic procedures [5,6]. These approaches enable significant control of severity and territory of cerebral injury. Additionally, rodent models have the added advantage of also providing complex genetic backgrounds for other stroke risk factors like diabetes or atherosclerosis [7,8]. However, numerous failures in human clinical trials of neuroprotectants, thrombolytics and other therapeutics have called into question the translatability of findings in rodent models to human patients. As a consequence of this failed translation, the Stroke Therapy Academic Industry Roundtable (STAIR) recommended the testing of therapies in a large animal model, such as the pig, before embarking on human clinical trials [9-11].

Rodent models have significant anatomical and physiological differences relative to humans [9,10]. The relatively large gyrencephalic human brain is markedly different in architecture to the lissencephalic rodent brain [12-14]. Gyrification of the human brain results in numerous folds (gyri and sulci) that allow for an increase in cortical surface area without a disproportionate increase in intracranial volume [15]. Gyrification and connectivity of neural fibers in the brain appear to have a direct relationship with mechanical forces exerted by neural fibers leading to changes in cortical folding [16-18]. Abnormalities in gyrification in humans have been directly correlated with diseases, such as schizophrenia, Alzheimer’s disease, William’s Syndrome, indicating a close relationship between form and function [19-21]. This suggests that deviations caused by conditions such as ischemic injury may lead to changes that are unique to the gyrencephalic brain.

The rodent brain is also significantly different than the human brain in white-gray matter composition. The rodent brain is composed of <10% white matter, while the human brain >60% white matter [12-14,22,23]. Recent studies have shown that white matter uniquely contributes to clinical deficits in stroke patients [23]. However, this is unsurprising as gray and white matter are fundamentally different at the cellular level. Structurally both compartments contain glia. However, white matter is mostly devoid of neuron cell bodies, dendrites and synapses, which are compartmentalized to the gray matter regions [24]. White matter is mostly composed of astrocytes, oligodendrocytes and myelinated and unmyelinated axons. The glia in white and gray matter compartments also behave differently due to functional requirements. For example, oligodendrocytes as a component of white matter produce a myelin sheath that covers 99% of axons to support the rapid and saltatory conduction of electrical signaling [24]. The loss of axon myelination results in slowing of conductive velocity and axonal degeneration, ultimately leading to brain atrophy and impaired cognitive, motor and sensory function. Ischemic injuries in both gray and white matter regions share basic response pathways, including cell death, excitotoxicity, immune and inflammatory responses, but there are also critical differences in pathogenesis between these compartments. The cellular composition of gray matter has led this compartment to have a higher metabolism and therefore more rapid oxygen and glucose consumption resulting in increased vulnerability to ischemia [25]. This increased vulnerability may lead to higher levels of damage and secondary injury relative to white matter. The composition, architecture and size of the brain all have a significant effect on the way ischemic stroke presents with respect to cell death, excitotoxicity, inflammatory, immune responses, hemorrhaging, edema and other factors [25-27]. These findings show the importance of utilizing a translational stroke model as similar to humans as possible for basic mechanistic studies and the development of novel treatments.

The gyrencephalic pig brain has long been recognized for its anatomical and physiological similarities to humans, making it an excellent candidate for translational studies of ischemic injury (reviewed by [28]). The pig brain is more comparable to the human brain in size, only being 7.5 times smaller, while the rodent brain is approximately 650 times smaller [28]. The size of the pig brain is thus comparable to typical non-human primate models [28]. When developing treatments such as cell therapies, size will be a considerable variable to account for as it will affect the number of cells to be transplanted, sites of injection, the ability of the graft to be vascularized and the distance axons must travel to form appropriate connections. In addition, both human and pig brains are composed of >60% white matter, suggesting the cellular mechanisms of injury and recovery are likely to be similar [29,30].

In this study we investigated a permanent middle cerebral artery occlusion (MCAO) model producing focal cerebral ischemia in Yucatan miniature pigs. Magnetic resonance imaging and histological evaluation of acute and chronic stages showed a robust and consistent stroke phenotype comparable to human patients.

## Materials and methods

All work performed in this study was done in accordance with the University of Georgia Institutional Animal Care and Use Committee guidelines.

### Pre-surgical and anesthetic protocol

Surgical induction of ischemic stroke injury was first optimized in 4 male landrace pigs and once optimized; this study was performed in 7 male Yucatan miniature pigs (5 years old; weighing between 80.9-104.5 kg with mean weight 93.6 kg).

All pigs were administered antibiotics 30 minutes prior to surgery (Ceftiofur sodium (Naxcel®; 4 mg/kg IM). Pre-induction analgesia and sedation was obtained using xylazine (5 mg/kg IM), butorphanol (0.2 mg/kg IM) and glycopyrrolate (0.01 mg/kg IM). Anesthesia was induced with intravenous propofol to effect and prophylactic lidocaine (0.5 to 1.0 mL of 2% lidocaine) was administered topically to the laryngeal folds to facilitate intubation. Anesthesia was maintained with 1.5% inhalational isoflurane (Abbott Laboratories) in oxygen. Artificial ventilation was performed at a rate of 8–12 breaths per minute with tidal volume of 5–10 ml/kg. With the animal positioned in sternal recumbency, a venous catheter was placed in the left aural vein for fluid therapy. During surgery, lactated ringers solution was administered at a rate of 5 ml/kg/hour. Heart rate was monitored by Doppler probe placement on the ventral tail artery. Rectal temperature was recorded every 15 minutes using a digital thermometer.

The head was tilted to the left 45 degrees and secured to facilitate a right MCA approach, utilizing gravity to lift the cerebrum away from the bony skull base during intracranial surgery. Hair between the eye and ear was shaved and the skin was prepared in a routine manner for sterile surgery using Betadine and 70% alcohol. The surgical site was draped in a standard fashion.

### Middle Cerebral Artery (MCA) occlusion induced ischemic injury

Injury was induced by performing a frontotemporal craniectomy with orbital rim ostectomy and zygomatic arch Resection. A curvilinear skin incision was performed, extending from just above the right orbit to an area just rostral to the auricle. The temporal fascia and muscle were incised and reflected with the skin flap. Following exposure of the branches of the superficial temporal artery and the associated vein, the vessels were occluded using high frequency bipolar cautery forceps; the insertion of the temporalis muscle was incised from below the zygomaytic arch and the muscle was subsequently elevated from the parietal bone. The periosteum overlying the zygomatic arch was incised and bluntly elevated away. The zygomatic arch was partially resected and the rostral aspect of the resection extended from the insertion point of the orbital ligament caudally 3-4 cm. This exposed the ventral aspect of the calvaria to the level of the orbital fissure. A surgical defect was generated in the exposed calvaria using a pneumatic drill and burr and extended using Kerrison rongeurs. The lateral portion of the roof of the orbit was rongeured away, while the orbital contents were protected with a hand held surgical retractor. The visible dura mater was incised and reflected dorsally as a flap. The arachnoid was opened at the distal portion of the MCA. Gentle retractor pressure using a spatula was exerted to create exposure of the basal cisterns so that cerebrospinal fluid (CSF) would be released, facilitating further brain exposure.

The MCA was located just distal to the Circle of Willis at its origin and was permanently occluded utilizing the bipolar electrocautery forceps. The exposed brain was then covered with a sterile biograft made of porcine small intestine submuscosa (MatriStem, ACell); the temporalis muscle was routinely reattached along the temporalis line and the skin was routinely re-apposed.

Banamine (1.1 mg/kg) and dexamethasone (4 mg each pig) were administered intramuscularly for postoperative pain and acute inflammation management during closing of the incision. Anesthesia was discontinued and pigs were returned to their pens upon extubation and monitored every 4 hours for the next 12 hours. Heart rate, respiratory rate, and temperature were recorded at each time point.

### Magnetic resonance imaging

Magnetic resonance imaging (MRI) was performed 24 hours and 90 days post-MCAO surgery on a GE 16-channel fixed-site Twin gradient Signa HDx 3.0 Tesla MRI system. Under anesthesia, MRI of the neurocranium was performed using a multichannel phase array spine coil, with the patient in dorsal recumbency. Standard multiplanar MR brain imaging series were acquired. These included T2-weighted (T2w), T2-weighted fluid attenuated inversion recovery (FLAIR), and T1-weighted (T1w) FLAIR, as well as diffusion-weighted imaging (DWI) series. DWI was acquired with b = 0 and b = 1000. DWI, apparent diffusion coefficient (ADC) maps and T1w-FLAIR images were evaluated using Osirix® software for presence of cerebral infarction and changes in cerebral hemisphere volume. Specifically, the volume of the ischemic area was manually derived from the ADC maps generated from DWI sequences. The ischemic area was defined by two levels of ADC number reduction, with the ADC number from the contralateral cerebral hemisphere providing normal ADC values. Ischemic areas, defined by those with 80% and 40% ADC values of normal, were manually traced on the sequential ADC images. Each area was multiplied by slice thickness to produce a volume of ischemic tissue. This method was chosen because it has been demonstrated to strongly correlate with histologically defined areas [31]. The cerebral hemisphere volume was determined through a similar process, whereby the cerebral hemisphere volume was quantified (excluding sulci and the lateral ventricular spaces) on T1w FLAIR images. The T2w FLAIR images were used for reference to differentiate areas filled with CSF and parenchymal areas of hyperintensity.

Immediately following the 90 days post-MCAO surgery MRI and while under anesthesia, all pigs were euthanized with a pentobarbital overdose (1 ml/4.5 kg).

### Neurological assessment

Neurological examinations adapted from those standardly performed on dogs were used to assess the pigs prior to the stroke injury and daily following injury. Examinations included assessments of menace response, pupillary light reflex, qualitative gait assessment, postural reactions, level of consciousness and behavior.

### Histology

Brains were removed within 1–2 hours of euthanasia and immersed in 10% buffered formalin. Following complete fixation, all surfaces of the brain were photographed with an in photograph ruler, and then approximately 5 mm wide transverse sections were made through the entire brain starting at the olfactory bulb. The caudal surface of each section was photographed with an in photograph ruler. Representative sections were taken to evaluate the infarct microscopically, routinely processed, embedded in paraffin, and stained with hematoxylin and eosin and/or luxol fast blue/periodic acid Schiff/hematoxylin. Additional sections were stained by immunohistochemistry for glial fibrillary acidic protein (GFAP; 1:4000; mouse; Biogenex), myelin basic protein (MBP; 1:500; mouse; Abcam), olig2 (1:200; rabbit; Genetex), and neurofilament (1:10,000; mouse; Biogenex). Heat induced antigen retrieval was performed for GFAP, MBP, and olig2 using citrate buffer at pH6 (DAKO). Detection was done using biotinylated anti-mouse (GFAP, MBP, neurofilament) or anti-rabbit (olig2) antibodies and a streptavidin label (4plus Streptavidin HRP or AP label; Biocare). For GFAP, olig2, and neurofilament a HRP label and DAB chromogen (DAKO) was used and for MBP, an AP label and Warp red chromogen (Biocare). All sections were lightly counterstained with hematoxylin. Histologic changes, including connective tissue proliferation, blood vessel density, astrocytosis/astrogliosis, macrophage infiltration, and lymphoplasmacytic inflammation, were evaluated for the leptomeninges over the infarct center, the center of the infarct andbordering reactive zone, and remote zones.

The area of the infarct, as viewed from the lateral surface, was measured using the Photoshop® software measurement tool and the in photograph ruler for calibration. The volume of the infarct was measured from transverse sections starting with the first section containing olfactory ventricle to the Section 5–10 mm from the caudal aspect of the cerebral hemisphere (occipital lobe). For each section, using the Photoshop measurement tool and in photograph ruler for calibration, the area (area of the parenchyma minus the area of the ventricle) of the right and left sides was determined separately and the area of each section was multiplied by the thickness of the section (5 mm). The sum of the volumes of each section was the overall volume of the hemisphere.

### Statistical analysis

Data was analyzed with SAS version 9.3 (Cary, NC) and statistical significances between groups were determined by two-way analysis of variance and post-hoc Tukey’s Pair-Wise comparisons. To determine the correlation between 24 hr ADC values and 90 day T1 FLAIR values, a Pearson Product–moment Correlation test was performed. Treatments where p-values were ≤0.05 were considered to be significantly different.

## Results

### Occlusion of the MCA results in ischemia and cytotoxic edema

**Figure 1:**
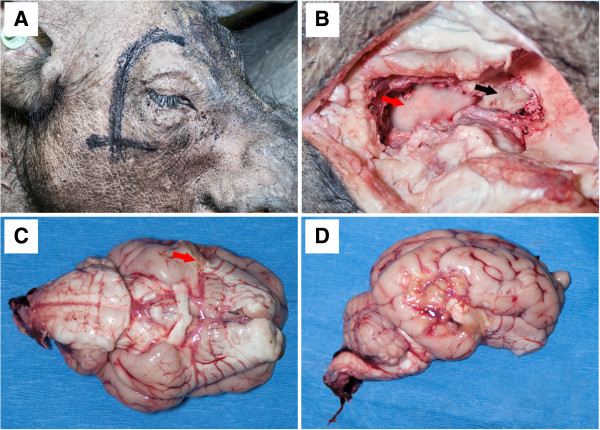
**Permanent MCA occlusion leads to significant infarction.** The vertical line marking on the animals head shows the line of incision with the horizontal line indicating the level of the zygomatic arch **(A)**. A portion of overlying zygomatic arch was resected, the temporalis muscle was elevated dorsally off the parietal bone (**B**, red arrow, lateral view). A window was generated in the exposed skull bone surface and the local dura mater exposed (**B**, black arrow). The proximal MCA was then permanently occluded (**C**, red arrow shows site of occlusion, ventral view) and resulted in infarction spanning the most caudal aspect of the frontal lobe and significant areas of the temporal lobe and portions of the parietal and occipital lobes (**D**, lateral view).

**Figure 2:**
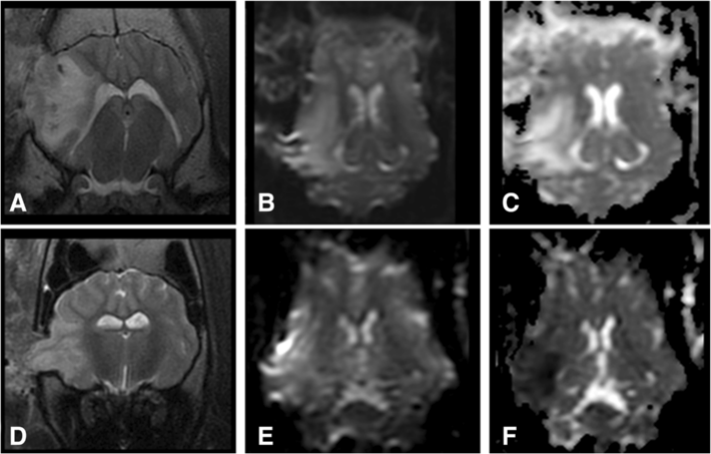
**ADC mapping is important for stroke discrimination.** T2-weighted transverse **(A & D)**, dorsal plane DWI **(B & E)** and ADC maps **(C & F)** are shown of animals that underwent stoke surgery. Animals that were unsuccessfully stroked **(A-C)** and successfully stroked **(D-E)** both had abnormal territorial parenchymal T2w hyperintensity in the right cerebral hemisphere in T2 **(A & D)** and DWI **(B & E)** images indicative of neural injury. However, the reduced territorial signal intensity on ADC maps seen in panel **(F)** is consistent with the restricted diffusion that is typical of infarctions, while this is not observed in panel **(C)**.

To optimize the MCAO surgery for the induction of stroke, male landrace pigs were used and once optimized this study was performed in male Yucatan miniature pigs. Ischemic stroke was induced by performing a frontotemporal craniectomy and cauterization of the MCA leading to permanent occlusion (Figure 1). Attempted cauterization of the MCA proximal to the Circle of Willis initially resulted in injury but not definitive ischemia. T2w (Figure 2A) and DWI (Figure 2B) MRI images had territorial hyperintense lesions indicative of an edematous injury. However, correlated ADC maps also had similar hyperintense regions indicative of vasogenic cerebral edema instead of cytotoxic edema typically associated with ischemic stroke (Figure 2C). The MCA exhibited significant branching making it challenging to cauterize each branch resulting in incomplete loss of blood flow; this is particularly true with this region of the brain possessing significant collateral blood flow (Figure 1C). Moving slightly distal to the Circle of Willis, the branches of the MCA were more readily identifiable. Cauterization of these more distal branches consistently led to hyperintense regions in T2w and DWI sequences, with corresponding hypointense areas on ADC maps (Figure 2D-F). Hypointensity on the ADC images confirmed areas of restricted diffusion, corresponding to cytotoxic edema indicative of ischemia. This demonstrated that distal cauterization of the MCA results in bona fide, repeatable ischemic stroke and not simply neural injury. Sham control surgeries were not performed as part of this study. However, sham control animals could be generated by performing the described surgery and excluding only the cauterization of the MCA from the procedure.

**Figure 3:**
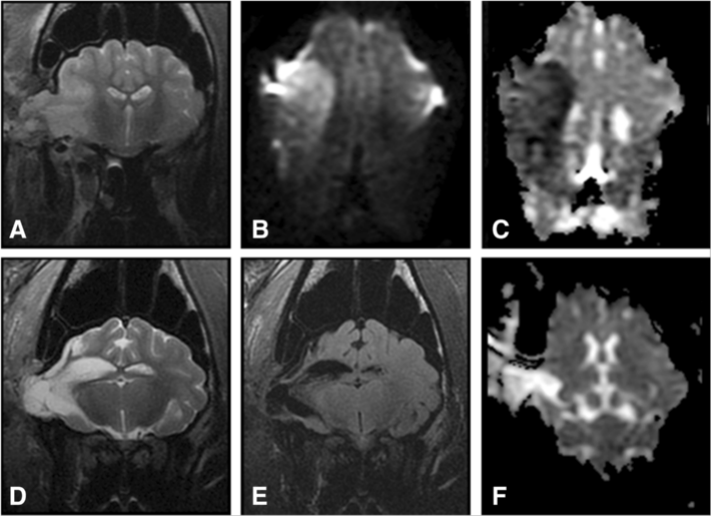
**Ischemic injury results in cerebral atrophy and aberrant brain remodeling.** T2-weighted transverse **(A & D)**, dorsal plane DWI **(B)**, dorsal ADC maps **(C & F)** and T2w-FLAIR **(E)** are shown from a pig with MR characteristics of a stroke at 24 hrs **(A**-**C)** and 90 days post **(D**-**F)**. At 24 hrs post-lesion, abnormal cerebral territorial hyperintense lesions were identified in 7 pigs in the T2w **(A)** and DWI **(B)** images, with territorial regions of restricted diffusion on the ADC maps **(C)**. At 90 days, marked cerebral cortical atrophy was present, with CSF filled cavities remaining at the prior site of infarction, consistent with hydrocephalus ex vacuo **(D**-**F)**.

At 24 hours post-MCAO, territorial hyperintense lesions were present in the T2w and T2-FLAIR images of all stroked pigs with lesions focused in the right temporal and piriform lobes (Figure 3A). The hyperintensity involved both the gray and white matter regions of these cerebral lobes. Lesions were most hyperintense at the junction between the cerebral gray and white matter of the coronal radiation. The hyperintensity radiated into the cerebral parenchyma. These hyperintense areas were hypointense on the T1w-FLAIR images. A mild mass effect was seen in association with the territorial lesions. This was characterized by compression of the right lateral ventricle and mild leftward deviation of the longitudinal fissure. No caudal transtentorial or foramen magna herniation was seen in any of the pigs, but all pigs had minor herniation through the craniectomy site. At 90 days post-MCAO surgery, significant cerebral cortical atrophy was present, characterized by enlargement of the right lateral ventricle or cavitated areas filled with CSF in the ischemic areas identified 24 hours post-MCAO (Figure 3D). The latter is consistent with hydrocephalus ex vacuo.

DWI and ADC maps were acquired for detection of restricted diffusion at 24 hours post-MCAO surgery, and were used to define ischemic areas (Figure 3B and C). DWI and ADC maps effectively defined territorial ischemic lesions, in contrast to the lesions in the Landrace pigs that did not have edema limited to the cytotoxic form (Figure 2C). The territorial areas of restricted diffusion commonly had heterogeneity in the degree of hypointensity seen in the ADC maps. The most hypointense areas were consistently seen in the tissue at the junction between the cerebral gray matter and the white matter of the coronal radiation. At 90 days post-MCAO surgery, significant areas of parenchymal loss and cavitation were evident, consistent with atrophy and hydrocephalus ex vacuo, respectively (Figure 3D and E). In addition, evidence of tissue ischemia (i.e. restricted diffusion) was no longer present on ADC maps (Figure 3F).

### MRI analysis shows significant tissue loss 90 days post-MCAO

**Figure 4:**
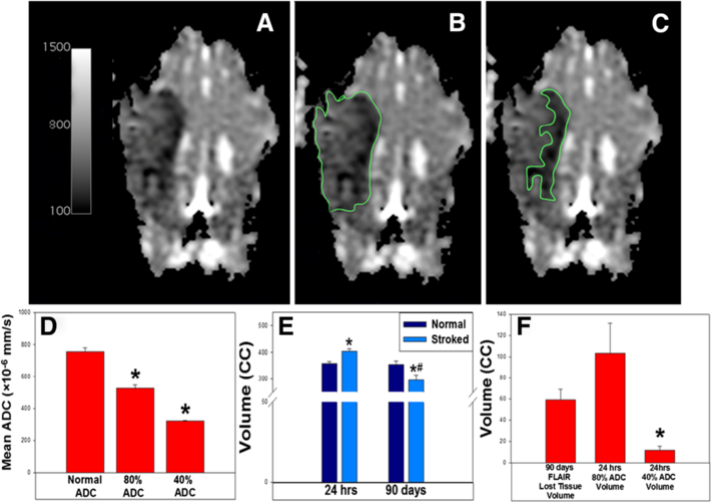
**Quantitative assessment of ADC and T1 flair images show ischemia, edema and atrophy.** ROI analysis was performed on ADC maps **(A)** with regions of 80% **(B)** and 40% **(C)** normal ADC being determined. Mean ADC value of normal control tissue of 755.00 ± 24.26 ×10−6 mm/s was significantly (*= significant difference at p< 0.05) higher than 80% and 40% ADC means of 525.43 ± 21.61 ×10−6 mm/s and 321.29 ± 2.83 ×10−6 mm/s respectively **(D)**. T1 FLAIR ROI analysis showed an increase in right (stroked) cerebral volume at 24 hrs (*= significant difference at p< 0.05 within time point) with mean right and left (normal) cerebral volumes of 404.17 ± 8.73 cc and 358.50 ± 7.16 cc respectively **(E)**. Between 24 hrs and 90 days, no significant difference was noted in the mean left cerebral volume (90d: 354.50 ± 1290 cc). The mean right cerebral volume at 90d, measuring 295.33 ± 20.07 cc was significantly (# = significant difference at p< 0.05 within brain hemisphere) decreased from left normal cerebral volume (354.50 ± 12.90 cc) at 90 days and the 24 hrs mean right cerebral volume (404.17 ± 8.73 cc). The volume of lost tissue (59.17 ± 10.06 cc), as quantified from the T1w-FLAIR was not significantly different from 80% ADC volume (103 ± 28.18 cc), while 40% ADC volume (11.50 ± 3.86) was significantly different (**F**;*= significant difference at p< 0.05).

Region of interest (ROI) analyses of ADC maps were performed to determine the territory of ischemic lesion at 24 hours (Figure 4). The entire territorial area of the ischemic lesion was measured as the region with ADC values 80% of the ADC values in the normal contralateral lobe (Figure 4B). The more hypointense areas on the ADC maps correlated to the regions with ADC values 40% of the normal ADC values (Figure 4C). ROI with 80% (525.43 ± 21.61 × 10^−6^ mm/s) and 40% (321.29 ± 2.83 × 10^−6^ mm/s) threshold had significantly (p-value < 0.05) lower ADC values than normal lobes (755.00 ± 24.26 × 10^−6^ mm/s; Figure 4D). The 80% ADC lesion region spanned a volume of 103 ± 28.18 cc and the 40% ADC lesioned region covered a volume of 11.50 ± 3.86 (Figure 4F). T1w FLAIR images were used to quantitate changes in tissue volumes at 24 hours and 90 days as well. A significant (p-value < 0.05) increase in the right cerebral volume was identified in the stroked hemisphere at 24 hrs post-MCAO, with mean right and left cerebral volumes of 404.17 ± 8.73 cc and 358.50 ± 7.16 cc, respectively. This increase in volume was attributed to edema (Figure 4E). The mean right cerebral volume at 90d was 295.33 ± 20.07 cc, which was significantly reduced relative to the left cerebral volume at 90 days (354.50 ± 12.90 cc) and the right cerebral volume at 24 hours (404.17 ± 8.73 cc). These cerebral volume changes showed a 59.17 ± 10.06 cc loss of tissue volume at 90 days in the right stroked cerebral hemisphere, based on the left normal hemisphere volume (Figure 4F). Between 24 hrs and 90 days, no significant difference was noted in mean left cerebral volume.

### 24 hour ADC map volumes correlate with loss tissue volumes at 90 days

ADC values at 24 hours may potentially be used to predict lost tissue volumes at 90 days. A Pearson Product–moment Correlation test was performed and demonstrated that 24 hour 80% ADC volume values (103 ± 28.18 cc) and lost tissue volume values based on T1w FLAIR (59.17 ± 10.06 cc ) were strongly positively correlated, with a Pearson correlation coefficient of 0.8141 at a p-value of 0.0489. The 40% ADC volume values did not significantly (p-value < 0.05) correlate with T1w FLAIR loss of tissue volumes. In addition, ANOVA and post-hoc Tukey’s T-test analysis showed there were no significant differences between lost tissue values determined by T1w FLAIR and 80% ADC volume, while 40% ADC volume significantly (p-value < 0.05) underestimated the loss of cerebral tissue (Figure 4F).

### Clinical assessment

Each pig demonstrated significant unilateral neurological deficits as soon as they were recovered from the anesthesia. These consisted of loss of contralateral menace, proprioception and motor deficits and circling toward the side of the lesion. Motor deficits included difficulty ambulating and observable weakness in the paretic limb. Profound mentation changes were observed in the first 24 hours. Recovery was noted to begin in all pigs within 72 hours and progressed continually for at least the first 30 days of the study.

### Gross histological examination demonstrates significant tissue loss in stroked hemisphere

**Figure 5:**
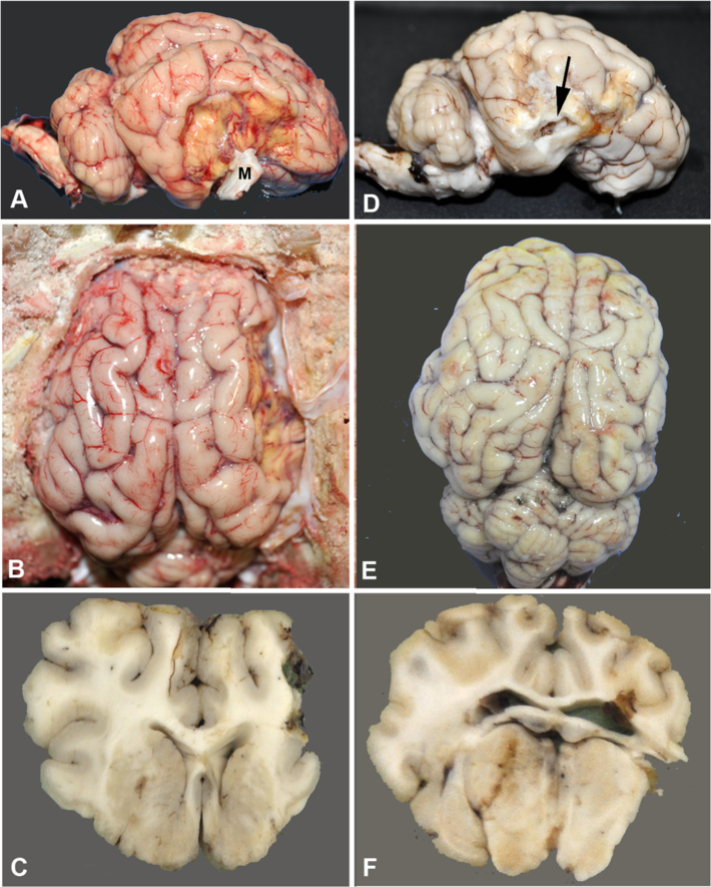
**Gross morphological changes in two representative stroked pigs. A**, **B** (in situ), and **C** are lateral and dorsal views and a transverse section through the infarct core of one pig, respectively. **D**, **E**, and **F** are from a second pig. The lateral area of the infarct cores are 7.04 cm^2^**(A)** and 9.42 cm^2^**(D)**. The core in **A** is well circumscribed and depressed with adherent meninges (M). The core in **D** is less circumscribed, slightly depressed and when the brain was removed adherent meninges pulled and disrupted a portion of thinned necrotic parenchyma (arrow). Note decreased vascular density around the core in both brains. From the dorsal surface, in both brains, the entire right hemisphere (stroked side) is smaller than the left. The stroke volumes are 14.14 cc **(B)** and 5.69 cc **(E)**. On transverse section, there is extensive loss of cortical and subcortical white matter on the right side in the first pig **(C)**. Less extensive tissue loss is present in second animal **(F)**, but the right lateral ventricle is dilated and communicates with the surface.

At day 90, all animals were sacrificed and brains were removed for gross and histological examination. Gross examination showed decreased parenchymal vasculature over the surface of the temporal and piriform lobes surrounding the infarct (Figure 5A and D). As viewed laterally, the center of the infarct primarily involved the temporal lobe and was variably depressed due to necrosis and tissue loss. The dura mater was typically adhered to the area of occlusion, which sometimes resulted in disruption of the cerebrum when the brain was removed (Figure 5D). The largest infarcts extended from the rostral suprasylvian sulcus rostrally to the caudal suprasylvian sulcus caudally, and from the middle suprasylvian sulcus dorsally to the lateral rhinal sulcus ventrally. In addition, the entire right hemisphere was grossly smaller than the left, due to diffuse atrophy (Figure 5B and E). In transverse sections, infarction also included the dentate gyrus ventrally and central portions of the occipital lobe caudally (Figures 5C and F). Atrophy of gyri remote to the infarct was evident as was atrophy of gray and white matter structures of the diencephalon, hippocampus and midbrain were also atrophied. Neuroparenchymal atrophy resulted in expansion of the lateral ventricles.

### Atrophy, glial scarring and macrophage infiltration in infarct region

**Figure 6:**
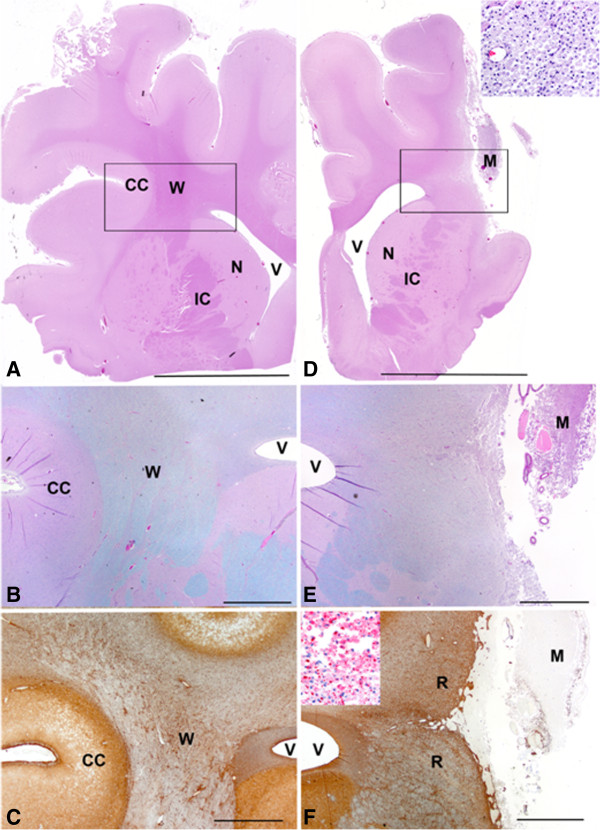
**Loss of cerebral cortex and macrophage infiltration at the infarct center.** Microscopic images of the unaffected brain **(A**, **B**, **C)** and affected brain **(D**, **E**, **F)** at the level of the infarct center. Box in **A** and **D** surrounds approximate area shown in **B-F**. **A** and **D**: Loss of cerebral cortex (CC) and underlying white matter (W) on the infarcted side is marked and much of the area is filled with foamy macrophages (M). Inset in **D** is a higher magnification of foamy macrophages. Note that the ventricle (V) is larger on the infarcted side and such structures as the caudate nucleus (N) and internal capsule (IC) are atrophied when compared to the contralateral side. **B** and **E** are higher magnification of the areas in the boxes from **A** and **D**. Loss of cerebral cortex (CC) and white matter (W) on the infarcted side is more evident and macrophages (M) can be seen filling the defect. **C** and **F**: The reactive zone (R) around the area of tissue loss showed increased GFAP staining due to astrocytosis and astrogliosis indicating glial scar formation. Inset in **F**: Macrophages (M) contained abundant myelin when stained for MBP seen as globular red staining. **A**, **D**: bar = 1 cm; **B**, **C**, **E**, **F**: bar = 2 mm. **A**, **D** Hematoxylin and eosin; bar = 1 cm. **B**, **E** Luxol fast blue/PASH, bar = 2 mm and **E**, **F** GFAP immunohistochemistry. Bar = 2 mm. Inset in **D**: Hemoxylin and eosin. Inset in **F**: MBP immunohistochemistry.

Microscopically, changes in the stroked right cerebrum could be divided into three areas: infarct center, reactive zone (area surrounding the infarct center) and remote zone (ipsilateral region distant from infarct). At the center of the infarct, there was extensive neuroparenchymal loss involving the cerebral cortex and underlying white matter with remnants of the neuroparenchyma filled with varying numbers of foamy macrophages (gitter cells) (Figures 6D-E). Foamy macrophages often contained myelin debris, as seen by MBP immunohistochemistry (Figures 6F). Lateral ventricles were also enlarged due significant atrophy of the neuroparenchyma (Figures 6D and E). These features were not observed in the normal hemisphere (Figures 6A-C).

**Figure 7:**
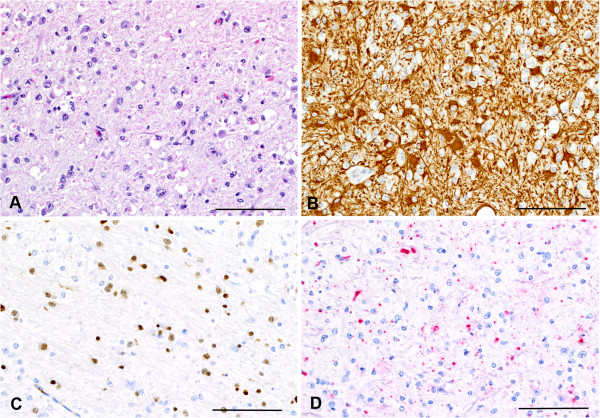
**Serial sections of reactive zone surrounding infarct show glial scar formation.** Hematoxylin and eosin stained sections show hypercellularity and loss of normal neuroparenchymal architecture in the reactive zone surrounding the infarct center **(A)**. GFAP staining in serial section showed increased cellularity due to increased numbers (astrocytosis) and size (astrogliosis) of astrocytes indicating glial scar formation **(B)**. Serial sections stained for Olig2 showing presence of oligodendrocytes (brown nuclear staining) with enlarged nuclei **(C)**. MBP staining (red) showing loss of myelin and remnant myelin is seen as globular staining rather than linear **(D)**. **B** and **C**: DAB chromogen with hematoxylin counterstain. **E**: Fast red chromogen with hematoxylin counterstain. Bars = 100 m.

Surrounding the center was a wide reactive zone where the neuroparenchyma was hypercellular, mostly due to varying degrees of astrocytosis and astrogliosis (glial scarring), confirmed with GFAP immunohistochemistry (Figures 6F and 7A and B). This zone had mild infiltration of macrophages often containing myelin debris detected by MBP staining, and mild microgliosis as determined by nuclear morphology (Figure 6F). Compared to the contralateral side, an increased number of oligodendroglia with enlarged nuclei were observed, as determined by olig2 immunohistochemistry (Figure 7C). MBP staining showed loss of myelin and remnant myelin was observed as globular staining rather than expected linear staining (Figure 7D). Reduced immunohistochemical staining for MBP and neurofilament, respectively, indicated decreased myelin and loss or atrophy of axons affecting white matter.

**Figure 8:**
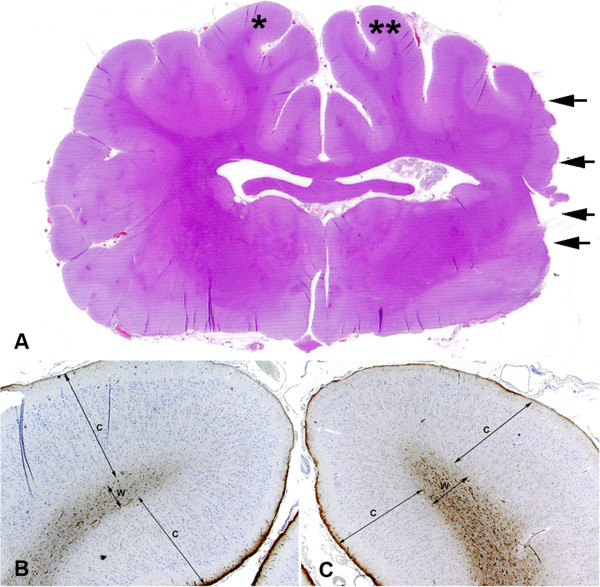
**Remote zone areas of the ipsilateral hemisphere display cortical thinning and white matter astrogliosis.** Transverse sections through the center of infarct (denoted by arrows) showed gyri away from the center of injury are thinner on the ipsilateral side **(A)**. Gyrus on unaffected side (*) has an overall diameter greater than the affected (**) side **(B**, **C)**. Outer and inner cortices **(C)** of the unaffected side are 1.95 mm and 1.54 mm, respectively and white matter (W) is 0.42 mm **(B)**. Gyrus on affected side has outer and inner cortices **(C)** of 1.61 mm and 1.43 mm, respectively, and white matter (W) of 0.54 mm **(C)**. While the overall diameter of the gyrus on the right infarcted side is less than the unaffected side, this is due to cortical thinning as the white matter on the affected side has an increased diameter due to astrocytosis and astrogliosis as seen by increased GFAP staining (brown) of the white matter.

Extending away from the reactive zone, there was remote neuroparenchymal atrophy encompassing the remaining gyri of the cerebral hemisphere, the hippocampus, structures of the diencephalon, and midbrain (Figure 8A). On the affected side, gyri were atrophied due to thinning of the cortex (Figure 8B and C) even though white matter of the corona radiata was expanded due to mild astrocytosis and astrogliosis as detected by GFAP immunohistochemistry.

### Comparative volumetric analysis of infarct region

Gross pathological measurement of the lesion aspect viewed from the lateral surface ranged from 4.26-9.42 cm^2^ with lesion volumes ranging from 4.22 cc to 14.14 cc. These volumes showed a loss of tissue that ranged from 11.2% to 35.3% with an average of 21.7 ± 3.83% relative to the normal contralateral hemisphere. From the areas of individual sections, atrophy of the entire right hemisphere was evident. For example, in sections at the level of the olfactory lobes (rostrally) and occipital lobes (most caudal), the area of the right brain was, on average, 6.9% and 15.2% less than the contralateral side, respectively.

It is challenging to compare infarct volumes determined by MRI and gross pathological measurements due to changes in brain morphology once removed from the calvarium and shrinkage caused by fixation. However, it is potentially possible to compare lost tissue volumes using these two methods by assessing percentages of tissue loss. Gross pathological measurements showed an average tissue loss of 21.7 ± 3.83%, while T1w FLAIR MRI assessment showed an average tissue loss of 19.3 ± 3.40%. These percentages were not statistically (p-value < 0.05) different from one another, suggesting that both methods gave comparable results in determining tissue loss.

## Discussion

In this study, we characterize a miniature pig MCAO model generated by a transcranial approach that demonstrates significant and consistent infarction with gray and white matter involvement. MRI analysis enabled the identification of true ischemic injury through the use of ADC maps and revealed significant cerebral swelling, cytotoxic edema at 24 hrs post-MCAO and loss of tissue in the infarct region at 90 days. Infarct regions demonstrated characteristic tissue loss involving up to 35% of the brain and spanning the frontal, temporal, parietal and occipital lobes. Ischemic injury led to dynamic macro- and microcellular changes in the brain, including loss of normal myelination of neurons, macrophage infiltration and glial scarring. These changes are consistent with ischemic stroke. These results suggest that this large animal pig model may be a robust system to study the pathophysiology of focal ischemic stroke and the development of diagnostics and treatments.

DW images have proven to be highly sensitive in the detection of ischemic stroke, sometimes within minutes of onset. However, the post-process ADC maps are critical for distinction of restricted diffusion, which is characteristic for an ischemic stroke. The loss of blood flow in an ischemic stroke leads to depletion of oxygen, ATP and metabolic collapse, with subsequent cellular membrane depolarization [32,33]. This results in cytotoxic edema, with intracellular swelling due to an influx of water into cells and a decrease in water diffusion in the affected brain parenchyma. This loss in diffusion is manifested as a hyperintense region in DWI. However, ADC maps are critical for correct assessment as DWI hyperintensities can also result from vasogenic edema caused by trauma or hemorrhage [34]. In addition, DWI is susceptible to artifacts, including T2 shine-through and receiver coil sensitivity-based intensity variation [33]. Previous studies of ischemic stroke in pigs utilized MRI to detect neural injury with T1w, T2w, FLAIR and DWI images [14,35]. However, they did not assess ADC maps of these animals. In this study, we showed that initial efforts resulted in vasogenic edema, suggestive of neurotrauma instead of ischemic stroke. This was likely the result of collateral blood flow reperfusing the site of injury and leading to increased extracellular fluid and vasogenic edema. Our surgical approach was adjusted so that cauterization of the MCA was more distal to the Circle of Willis eliminating collateralization and resulting in cytotoxic edema. In addition, MCA branching proximal to the Circle of Willis was found to be complex and highly variable between animals. In some cases, branch points began almost immediately adjacent to the Circle of Willis and branching ranged from 3 to 6 branches making it challenging to identify and cauterize all MCA branches. Cauterization more distal resulted in better MCA branch identification and more consistent ischemic infarction. Once this surgical approach was optimized, it lead to 100% (7 out of 7 Yucatans) stroke efficiency that could be regularly identified by marked cytotoxic edema in hypointense regions of ADC maps.

MRI assessment of stroke also proved to be a highly predictive tool in the pig model, as it has been shown in human patients. The T1w FLAIR MRI analysis at 90 days showed an average loss of brain tissue of 59 cc, which was highly correlative to tissue loss estimated by the 80% ADC volume at 24 hrs. Infarct size was confirmed by gross histological measurements. Similar studies have been performed in human patients and further refinement of MRI technology has lead to improved discrimination between penumbra or damaged tissue that is potentially salvageable, and core stroke regions or tissue that is typically completely lost, [32,33]. Further studies are needed to expand MRI data (e.g. diffusion tensor imaging (DTI) and perfusion imaging) in this pig model to further correlate the lesion seen in pigs to that seen in humans with strokes. Respectively, these techniques would better define the regions of brain parenchyma affected, such as white versus gray matter, and potentially the different zones seen histologically in a larger morphologically similar brain. MRI analysis, supported by histology, also showed standard evolution of ischemic injury, initiating with edema and cerebral swelling with both gray and white matter involvement followed by cerebral atrophy and in some cases foci of hydrocephalus ex-vacuo. The reactive zone showed infiltration of macrophages, cells that play a complex role in ischemic injury. These cells serve a positive role by removing cellular debris and in a M2 phenotype (also known as “alternatively activated”) can produce neuroprotective cytokines. They also can have a negative impact and can lead to increased secondary injury in the M1 phenotype (also known as “classically activated”) by enhancing the local proinflammatory response leading to increased cell death and lesion size [36,37]. A number of neuroprotectants have been developed to mitigate this secondary injury mediated in part by macrophages with limited success in Phase III clinical Trials [22]. It has become a general belief in the stroke community that this failure to translate to humans is in part due to the development of treatments in animal models that are dissimilar to humans in neuroanatomy and physiology [9-11]. Development of treatments in large animal models with gyrencephalic brains and similar gray-white matter composition to humans, such as the pig, would likely reduce the number of therapeutics that are destined to fail in human clinical trials.

The surgical approach utilized in this model had several advantages with one being that it is a relatively quick surgery taking less than 2 hrs from initial skin incision to final closure with sutures. This procedure also resulted in low morbidity with only one animal being lost post-surgery. Similar to a recent report by Imai et al. [35], our non-transorbital approach did not result in optic nerve damage, loss of vision or enucleation of an eyeball, which has been observed in transorbital approaches [38,39]. Enucleation would be a significant confounding variable for future motor function and behavioral studies, as it would alter depth perception and potentially limit the speed at which tasks can be performed [38,39]. However, there are some limitations to our surgical approach. A loss of appetite, or reduced feeding behavior in animal models, is one of the most common and measurable changes in stroke patients [40,41]. Loss of interest in eating is a potential sign of depression. The reported surgical approach leads to damage to the temporalis muscle, important for mastication, and partial resection of the zygomatic arch and may cause reduced feeding behavior. In this study, we found that 57% (4 out of 7 Yucatans) of Yucatans were able to eat unaided within 72 hrs of surgery. This included the sedated pigs in the initial post-MCAO 24 hr MRI time period where the animals had limited feeding opportunities and/or were fasted in preparation for general anesthesia for the post-MCAO MRI. Two (29%, 2 out of 7) of the remaining animals began to eat unaided within two weeks, while the remaining animal (14%, 1 out of 7) had to be sacrificed due to inability to recover. As with all transcranial approaches, this procedure does result in a cranial window that can function as a decompression zone. Minimal herniation through this craniotomy window was identified in the MR images at early time points. However, the optimized visual window provided by this approach enables clear identification of MCA branches, which led to improved reproducibility. Infarct reproducibility has been a major issue in studies where surgical approaches do not allow for optimum visibility [42]. In addition, the transcranial approach was used in this study as the pig has a rete mirabile, a complex and dense network of blood vessels, in the skull. The rete mirable limits the use of other approaches (e.g. embolic, catheter, ligation) for inducing ischemia due to blood vessel collateralization within the rete mirable or the inability to pass an embolus, catheter or the like beyond the rete mirable. Animals also showed functional deficits including limb weakness and impaired ambulation. Previous work by our group showed that a novel gait analysis system could be utilized for more refined and quantitative detection of changes in gait in biomedical pigs, which should be employed in future studies [43].

## Conclusion

In this study, we develop and characterize a pig ischemic stroke model with significant similarities in neuroanatomy and physiology to humans. The optimized surgical procedure resulted in consistent infarction that led to ADC-map verified cytotoxic edema, indicating true ischemic stroke. Ischemic injury showed classical evolution in both gray and white matter tissue, including infarction, macrophage infiltration, loss of structural elements and glial scarring. This pig MCAO ischemic stroke model provides an excellent opportunity to better understand stroke in a human-like system and to develop novel diagnostics, devices and therapeutics that can potentially be more easily translated to human patients.

## Competing interests

The authors declare that they have no competing interests.

## Authors’ contributions

SRP designed studies, performed surgeries, performed MRI and drafted manuscript. SPH designed studies, performed and interpreted MRI data and drafted manuscript. EWH designed studies, performed and interpreted histology and drafted manuscript. KJD assisted in data collect, data analysis and interpretation and drafted manuscript. CRD assisted in data collect, data analysis and interpretation and drafted manuscript. HAK assisted in data collect, data analysis and interpretation and drafted manuscript. ELW assisted in data collect, data analysis and interpretation and drafted manuscript. ELW assisted in data collect, data analysis and interpretation and drafted manuscript. AVL assisted in data collect, data analysis and interpretation and drafted manuscript. VWL assisted in data collect, data analysis and interpretation and drafted manuscript. SLS assisted in study design, data analysis and interpretation. WDH assisted in study design, data analysis and interpretation. DCH assisted in study design, data analysis and interpretation. FDW designed studies, data collection, analysis and interpretation, statistical analysis and drafted manuscript. All authors read and approved the final manuscript.
